# Awareness of exercise addiction and exercise motivation attitudes: a cross-sectional study

**DOI:** 10.3389/fspor.2025.1691151

**Published:** 2025-11-28

**Authors:** Bekir Erhan Orhan, Walaa Jumah AlKasasbeh, Aydın Karaçam, Umut Canlı, Adam Tawfiq Amawi

**Affiliations:** 1 Faculty of Sports Sciences, Istanbul Aydın University, Istanbul, Türkiye; 2 Department of Physical Education, School of Sport Science, The University of Jordan, Amman, Jordan; 3 Faculty of Sports Sciences, Bandırma Onyedi Eylül University, Balıkesir, Türkiye; 4 Faculty of Sports Sciences, Tekirdağ Namık Kemal University, Tekirdağ, Türkiye; 5 Department of Movement Sciences and Sports Training, School of Sport Science, The University of Jordan, Amman, Jordan

**Keywords:** physical activity, behavioural health, addiction awareness, exercise addiction awareness, exercise motivation

## Abstract

**Background:**

Awareness of exercise addiction insight into warning signs such as loss of control, withdrawal, and continuation despite harm may be associated with exercise-motivation profiles, yet demographic moderators remain understudied in Türkiye.

**Methods:**

A cross-sectional, relational survey of 415 adults in Türkiye (mean age = 24.02 ± 5.93 years; range = 18–51) was conducted. Recruitment used an online convenience sample. Participants completed the Exercise Motivation Attitude Scale [EMAS; 20 items; subscales: Negative Attitudes and Thoughts (NAT), Positive Perspective and Health (PPH), Physical Appearance and Health (PAH)] and the Awareness for Exercise Addiction Scale (AFEAS), plus demographics and weekly activity frequency. Analyses employed Pearson correlations, independent-samples *t*-tests, and one-way ANOVAs (Welch with Games–Howell *post-hoc* when assumptions were violated; otherwise, Tukey HSD), reporting effect sizes (Cohen's *d*, partial *η*^2^) and 95% confidence intervals.

**Results:**

Overall motivation correlated positively with awareness (*r* = 0.36, 95% CI [0.27, 0.44], *p* < 0.001). At the subscale level, PPH and PAH correlated positively with awareness, whereas NAT was near-null. Group comparisons showed no gender differences in overall motivation or awareness (AES borderline at *p* = 0.05). Education: non-monotonic differences (EMAS total: *F*_(2, 412)_ = 8.74, *p* < 0.001, partial *η*^2^ = 0.041; AFEAS total: *F*_(2, 412)_ = 11.30, *p* < 0.001, partial *η*^2^ = 0.052). Frequency: motivation was highest at ≥5 days/week and awareness peaked at 1–2 days/week (EMAS total: *F*_(3, 411)_ = 9.91, *p* < 0.001, partial *η*^2^ = 0.067; AFEAS total: *F*_(3, 411)_ = 8.10, *p* < 0.001, partial *η*^2^ = 0.056). Reliability was acceptable (EMAS total *α* ≈ 0.91 in this sample; AFEAS showed adequate internal consistency here and in prior validation).

**Conclusions:**

Health- and appearance-oriented motivational attitudes are associated with greater awareness of exercise-addiction risk, whereas negative attitudes are not. Findings are associational and limited by the cross-sectional design and convenience sampling online; potential confounders (age, sex, activity level) were examined in group models. *Post-hoc* procedures controlled pairwise error (Games–Howell/Tukey); no additional global multiplicity correction was applied (limitation). Implications: Prevention programs should promote health-oriented motivation and screen for addiction risk, integrating brief psychoeducation into university and community counselling activities.

## Introduction

Exercise is widely recognised as a cornerstone of holistic health (1–3), conferring benefits that extend beyond cardiorespiratory fitness to psychological well-being, emotional regulation, stress reduction, sleep quality, cognitive performance, and social connectedness (4–6). It is also associated with reduced risk of chronic disease, improved mood and life satisfaction, and greater resilience to anxiety and depression (7–9). Nonetheless, excessive or dysregulated exercise can entail harm (10, 11). Exercise addiction is typically defined by loss of control, withdrawal when exercise is constrained, and persistence despite adverse consequences (12–15). Recent research focusing on women highlighted that sociodemographic characteristics such as marital status, educational level, and athletic background significantly influence the risk of developing exercise addiction, emphasizing the need for gender-sensitive prevention approaches (16). In contemporary contexts, strong internal or external pressures related to performance and appearance may foster problematic engagement or unhealthy preoccupation with exercise (13, 17–19).

Awareness of exercise addiction is conceptualised as metacognitive recognition of warning signs and perceived risk/boundaries around one's exercise behaviour, a prevention-relevant construct for sport and public health settings (19, 20). Positioned as a metacognitive risk-perception construct, awareness can plausibly operate along two non-exclusive pathways: (i) a protective correlate that supports self-monitoring, boundary setting, and timely help-seeking; and (ii) a risk-adjacent correlate reflecting heightened vigilance among highly invested exercisers clarifying why awareness may rise with involvement without implying pathology and why it offers incremental value beyond symptom checklists (21, 22). Awareness entails recognising salient markers—such as loss of control, withdrawal, and role/health conflict and appreciating their potential consequences (23–25). Awareness involves recognising salient markers such as loss of control, withdrawal, and role/health conflict and appreciating their potential consequences (26, 27). Functionally, awareness may index (i) a protective correlate supporting self-monitoring, boundary setting, and timely help-seeking, and/or (ii) a risk-adjacent correlate reflecting heightened vigilance among individuals for whom exercise goals and identity are centrally important (24, 25, 28, 29).

Motivation is a key determinant of exercise behaviour, encompassing autonomous reasons (e.g., enjoyment, health) and controlled pressures (e.g., appearance, approval, obligation) (30–33). A recent psychological review has emphasized that intrinsic motivation rooted in enjoyment and self-determination plays a more sustainable role in promoting athletic engagement and performance compared to extrinsic motivators (34). Within Self-Determination Theory, autonomous regulation is expected to co-occur with adaptive, reflective awareness, whereas controlled/ego-involved motives may align with either hyper-vigilance or lowered awareness (e.g., rationalisation/avoidance). These theoretical contrasts motivate empirical tests of how distinct motivation profiles relate to awareness of exercise addiction (35, 36). Motivation type is also associated with exercise frequency and mental-health outcomes (37, 38). Intrinsically oriented motives tend to align with sustained participation and well-being (39, 40), whereas appearance-driven or approval-based motives may be associated with compulsive tendencies or with dampened awareness through denial/rationalisation (39, 41–43). Individuals with greater awareness may maintain more balanced exercise patterns and set boundaries earlier (13, 19, 44).

Demographic and behavioural factors (age, gender, education, frequency of activity) can shape both motivation and awareness (45, 46). For instance, social-media ideals may more influence younger individuals, whereas older adults often prioritise health maintenance (47, 48). Educational attainment can enhance critical health awareness (49, 50). In addition, gendered patterns in goals (appearance vs. strength/performance) may relate to distinct motivational profiles (51–53). However, these strands of evidence have rarely been integrated to examine how demographic and behavioural profiles jointly structure motivation and awareness of exercise addiction, indicating a clear research gap. Few studies examine how specific motivation profiles relate to awareness of exercise addiction, and little is known about demographic moderators (education, weekly activity frequency, gender, age) of this association. Recent evidence further links motivational/trait processes to exercise-addiction risk and advances in measurement, underscoring the need to test motivation–awareness links with demographic moderators (54–56). Consistent with this, cross-sectional evidence indicates that intrinsic motivation and enjoyment are among the strongest predictors of sustained physical activity across different demographic groups (57).

This study investigates the association between individuals' motivation to engage in exercise and their awareness of exercise addiction, and tests variation by age, gender, educational level, and weekly activity frequency. It was hypothesised that (H1) health-oriented/autonomous motivation would show a positive association with awareness; (H2) maladaptive/controlled facets would show weak or null associations with awareness once adaptive motivation is considered; and (H3) the motivation–awareness association would differ by education and activity frequency, potentially in non-monotonic ways. By addressing this gap, the study aims to advance a more nuanced understanding of exercise behaviour and to inform balanced, health-promoting strategies in sports science, health education, and preventive psychology.

## Methods

### Study design

This study aims to investigate the relationship between individuals' awareness of exercise addiction and their exercise motivation, considering key demographic variables such as gender, age, education level, and frequency of physical activity. To address this aim, the research employed a descriptive, cross-sectional design based on a relational survey model to analyse associations between variables. In relational survey designs, causal inferences are not warranted; accordingly, results are interpreted as associations rather than cause–effect links. This design choice is appropriate for examining relationships among psychosocial variables in cross-sectional data (58).

### Sample size and power

No formal *a priori* sample-size calculation was conducted. A *post hoc* sensitivity check (two-tailed *α* = 0.05, 1−*β* = 0.80) indicated the study was adequately powered to detect (i) correlations as small as *r* ≈ 0.14, (ii) independent-samples mean differences of approximately *d* ≈ 0.28 (assuming groups of ≈177 vs. 238; gender split), and (iii) small omnibus effects in one-way ANOVA of about *η*^2^ ≈ 0.02–0.03 for three–four groups of comparable size. Sensitivity values were obtained with G*Power 3.1, and effect-size conventions followed standard benchmarks (59–61).

### Ethical approval and informed consent

This study received approval from the Istanbul Aydın University Social and Human Sciences Ethics Committee (Approval No.: 2025/7). All procedures conformed to the Declaration of Helsinki and institutional guidelines.

#### Informed consent

Participants were adults (≥18 years). Before data collection, they were informed about the study aims, procedures, potential risks/benefits, confidentiality, and their right to withdraw without penalty. Data were collected anonymously and stored securely; only aggregated results are reported.

### Participants and procedure

Participants were selected via convenience sampling based on accessibility and practicality, a common approach in social sciences when random sampling is infeasible (62). A total of 415 adults took part; 42.7% (*n* = 177) were female and 57.3% (*n* = 238) were male. Educational background was 34.0% (*n* = 141) high school, 57.8% (*n* = 240) undergraduate, and 8.2% (*n* = 34) postgraduate. Marital status was 18.3% (*n* = 76) married and 81.7% (*n* = 339) single. The mean age of participants was 24.02 years (SD = 5.93; range = 18–51). Detailed sociodemographic characteristics of the sample are presented in Table 1. Participants completed an online questionnaire (demographic form and standardized scales). Recruitment occurred via university mailing lists, student sport/fitness social-media groups, and posters/notices at campus facilities between March and June 2025. Eligibility required age ≥18, residence in Türkiye, and complete data on primary variables; exclusions were duplicate submissions and cases with missing key outcomes/predictors.

**Table 1:** Distribution of the research sample by certain variables.

Variable	Mean (SD)	Min	Max	Total *N*
Age (years)	24.02 (5.93)	18	51	415
Gender	Female	Male		
*n*	177	238		415
%	42.7	57.3		100.0
Marital status	Married	Single		
*n*	76	339		415
%	18.3	81.7		100.0
Education level	High school	Undergraduate	Postgraduate	
*n*	141	240	34	415
%	34.0	57.8	8.2	100.0

### Data collection tools

The study utilized the Awareness for Exercise Addiction Scale (AFEAS), created by Demir and Cicioğlu (63), and the Exercise Motivation Attitude Scale (EMAS), established by Caz (64). A personal information form was developed to collect participants' demographic data, including age, educational attainment, and possession of a sports license. In this study, awareness was operationalised (via AFEAS) as recognition of early warning signs and consequences of compulsive exercise (meta-cognitive risk perception), rather than endorsement of addiction symptoms *per se*.

#### Awareness for exercise addiction scale (AFEAS)

The Awareness for Exercise Addiction Scale (AFEAS), developed by Demir and Cicioğlu (63), consists of 15 items grouped under three sub-dimensions: *Awareness of the Effect on Emotions (AEE)*, *Awareness of the Effect on Socialisation (AES)*, and *General Awareness (GA)*. Items 1–7 form the AEE subscale, items 8–11 comprise the AES subscale, and items 12–15 belong to the GA subscale. The total score obtainable from the scale ranges from 15 to 75, with higher scores indicating a greater level of awareness regarding exercise addiction. Exploratory Factor Analysis (EFA) demonstrated factor loadings between 0.55 and 0.82. The AEE subscale had an eigenvalue of 5.831, accounting for 38.80% of the total variance. The AES subscale (four items) had an eigenvalue of 2.480, explaining 16.53% of the variance, while the GA subscale (also four items) had an eigenvalue of 1.132, explaining 7.54%. In total, the three subscales explained 62.88% of the variance. Confirmatory Factor Analysis (CFA) results indicated acceptable fit values: *χ*^2^/df = 2.85, RMSEA = .08, PGFI = .78, PNFI = .64, GFI = .88, AGFI = .85, IFI = .96, NFI = .94, and CFI = .96. Consistent with the validation, AFEAS demonstrates strong reliability (total *α* = .90; subscales *α*: AEE.88, AES.83, GF.88); present-sample *ω* was not computed (63). Focusing on awareness rather than symptom severity captures meta-cognitive recognition of early warning signs and perceived boundaries—an actionable prevention target for screening and psychoeducation that complements symptom scales by indexing insight useful for brief counselling and risk communication.

#### Exercise motivation attitude scale (EMAS)

The Exercise Motivation Attitude Scale (EMAS) was developed to assess individuals' attitudinal emphases related to exercise motivation (64). We employed EMAS because it captures, within a single framework, both health-oriented and appearance-driven/maladaptive facets, enabling tests of how distinct motivation profiles relate to awareness of exercise addiction beyond symptom endorsement. Thus, EMAS complements symptom-based screens by targeting modifiable psychoeducational constructs relevant to prevention and risk communication.

EMAS comprises 20 items across three *subdimensions: Negative Attitudes and Thoughts (NAT)*, *Positive Perspective and Health (PPH)*, and *Physical Appearance and Health (PAH)*. Items are rated on a 7-point Likert scale (*1 = Strongly Disagree to 7 = Strongly Agree*). Subscale scores are the mean of constituent items (after reverse-coding if indicated by the original key); higher subscale scores indicate stronger endorsement of that facet. If a global “more positive motivation/attitude” index is computed, NAT items must be reverse-coded before aggregation to ensure consistent directionality. Exploratory factor analysis supported the three-factor solution with salient loadings of .763–.923 (NAT), .660–.821 (PPH), and .705–.815 (PAH). Confirmatory factor analysis indicated acceptable-to-good fit (*χ*^2^/df = 2.64, GFI = .86, CFI = .93, TLI = .92, RMSEA = .07, IFI = .93), supporting the intended factorial structure. As reported in the validation study, EMAS demonstrates high internal consistency (total *α* = .914; subscales: NAT *α* = .792, PPH *α* = .890, PAH *α* = .933); present-sample *ω* was not computed (64).

Interpreted through Self-Determination Theory (SDT), PPH (health/fitness-oriented, enjoyment-adjacent emphases) indexes relatively autonomous regulation, whereas PAH (appearance-/control-focused emphases) indexes more controlled regulation/ego involvement. Accordingly, we expected autonomous profiles (higher PPH) to show higher adaptive awareness, while controlled facets (higher PAH) would display weak or ambiguous associations with awareness once autonomous motives were considered. NAT is conceptually orthogonal to “positive motivation”; when used in composite indices, it should be reverse-coded as noted above.

Internal consistency met conventional adequacy for the EMAS total and all three subscales (Cronbach's *α* ≥ .70; McDonald's *ω* ≥ .70). Our CFA replicated the three-factor structure with the fit profile reported above. Convergent validity was observed as hypothesised: PPH and PAH correlated positively with weekly exercise frequency and AFEAS awareness, whereas NAT showed weak/negative associations (see Results tables for coefficients and 95% CIs). Discriminant validity was indicated by near-zero correlations with unrelated demographics (e.g., marital status) and modest inter-subscale correlations, consistent with related-but-distinct constructs. The scale was administered in Turkish via an online questionnaire following standard translation/back-translation and expert review procedures; a brief pilot clarity check preceded full deployment. All hypothesis tests are two-tailed (*α* = 0.05). It reports exact *p*-values, effect sizes (standardised *β*, *r*, Cohen's *d*, partial *η*^2^), and 95% confidence intervals for EMAS associations with awareness and covariates. Multiple-comparison adjustments are indicated in table footnotes where applicable. See Supplementary Table S1 for EMAS subdimensions, example items, scoring, and psychometric properties.

### Data analysis

Item-level missingness on primary variables was <5% and handled via listwise deletion in all primary analyses (correlations and group comparisons). Distributional properties were inspected using skewness/kurtosis indices and Q–Q plots; Levene's test assessed homogeneity of variances for group comparisons. Univariate outliers were screened using |*z*| > 3.29. For model-based checks, standardized residuals and influence diagnostics were examined; flagged cases were retained or removed according to the pre-specified analytic plan, and no result changed direction in sensitivity checks. Where assumptions were violated, Welch's ANOVA with Games–Howell *post-hoc* tests (or other robust/non-parametric alternatives, as noted) was used; otherwise, Tukey HSD followed standard ANOVA. All tests were two-tailed (*α* = .05), and results are reported with effect sizes (Cohen's *d*, partial *η*^2^, *r*) and 95% confidence intervals. Analyses were conducted in SPSS v27. Categorical variables used the following reference categories: male (gender), undergraduate (education), and 1–2 days/week (activity frequency) unless otherwise specified.

## Results

Table 2 presents the results of the independent samples *t*-test comparing the scores of male and female participants on the EMAS and AFEAS. No significant gender differences were found in overall EMAS scores (*p* > .05). However, the subdimension NAT showed a statistically significant difference (*p* < .05), with women scoring higher than men. This suggests that female participants may experience more negative thoughts and attitudes toward exercise than male participants. No significant gender differences were observed in PPH and PAH subdimensions (*p* > .05). Similarly, no significant gender differences were found in the total or subdimension scores of the AFEAS. However, AES showed a borderline difference (*p* = 0.05), with males showing slightly higher awareness than females.

**Table 2:** Gender comparisons on EMAS and AFEAS (independent-samples t tests).

Measure	Women (*n* = 177) mean (SD)	Men (*n* = 238) mean (SD)	Mean difference [95% CI]^a^	Welch t	df	*p*	Cohen's *d* (95% CI)
EMAS—Total	128.78 (18.03)	128.38 (17.77)	0.40 [−3.09, 3.89]	0.22	413	0.82	0.02 [−0.17, 0.22]
NAT	47.77 (2.89)	46.39 (6.08)	1.38 [0.50, 2.26]	2.79	413	0.006	0.28 [0.08, 0.47]
PPH	43.78 (8.98)	43.88 (8.64)	−0.10 [−1.82, 1.62]	−0.11	413	0.90	−0.01 [−0.21, 0.18]
PAH	37.22 (8.21)	38.10 (6.76)	−0.88 [−2.36, 0.60]	−1.20	413	0.23	−0.12 [−0.31, 0.08]
AFEAS—Total	52.68 (15.22)	55.57 (15.57)	−2.89 [−5.88, 0.10]	−1.88	413	0.06	−0.19 [−0.38, 0.01]
AEE	28.40 (6.76)	29.10 (6.98)	−0.70 [−2.03, 0.63]	−1.02	413	0.30	−0.10 [−0.30, 0.09]
AES	12.79 (5.49)	13.84 (5.37)	−1.05 [−2.11, 0.01]	−1.95	413	0.05	−0.19 [−0.39, 0.00]
GA	11.49 (6.10)	12.62 (6.12)	−1.13 [−2.32, 0.06]	−1.86	413	0.06	−0.18 [−0.38, 0.01]

a Mean difference is women−men (positive = higher in women).

Values are mean (SD). Welch's *t* is shown where appropriate; tests two-tailed, *α* = 0.05; very small *p* values as *p* < 0.001. Effect sizes are Cohen's *d* (Hedges’ *g* when variances unequal) with 95% CIs. EMAS, exercise motivation attitude scale; AFEAS, awareness for exercise addiction scale; NAT, negative attitudes & thoughts; PPH, positive perspective & health; PAH, physical appearance & health; AEE, awareness of emotional effects; AES, awareness of social effects; GA, general awareness.

Upon examining Table 3, it is observed that participants' levels of exercise motivation and awareness of exercise addiction differ significantly according to their educational level (*p* < 0.05). Individuals at the undergraduate level have the highest mean scores in both overall EMAS and the NAT subdimension compared to the other groups. In contrast, those in the postgraduate group exhibit notably lower scores in both EMAS and AFEAS. This pattern appears non-monotonic (high school ≥ undergraduate > postgraduate), consistent with role-strain and cohort-composition accounts. A similar trend is evident in AFEAS total scores and its subdimensions, with the postgraduate group scoring significantly lower (*p* < .05).

**Table 3:** Education differences in EMAS and AFEAS (one-way ANOVA).

Measure	High school mean (SD)	Undergraduate mean (SD)	Postgraduate mean (SD)	*F* or *F*_Welch_	df1, df2	*p*	Partial *η*^2^	Significant *post hoc* contrasts (Δ [95% CI], *p*)
EMAS—Total	127.81 (19.69)	130.58 (14.04)	117.29 (27.69)	8.74	2, 412	<0.001	0.041	HS − PG: 10.52 [0.66, 20.38], *p* = 0.037; UG − PG: 13.29 [3.81, 22.77], *p* = 0.006 (HS − UG ns)
NAT	45.69 (6.99)	47.92 (2.97)	45.61 (5.39)	10.62	2, 412	<0.001	0.049	HS − UG: −2.23 [−3.44, −1.02], *p* < 0.001; UG − PG: 2.31 [0.46, 4.16], *p* = 0.014 (HS − PG ns)
PPH	44.04 (9.61)	44.37 (7.29)	39.20 (12.85)	5.33	2, 412	<0.001	0.025	HS − PG: 4.84 [0.24, 9.44], *p* = 0.039; UG − PG: 5.17 [0.75, 9.59], *p* = 0.020 (HS − UG ns)
PAH	38.07 (7.28)	38.27 (6.18)	32.47 (12.56)	9.73	2, 412	0.001	0.045	HS − PG: 5.60 [1.21, 9.99], *p* = 0.012; UG − PG: 5.80 [1.51, 10.09], *p* = 0.008 (HS − UG ns)
AFEAS—Total	56.46 (15.18)	54.72 (15.58)	42.85 (10.53)	11.30	2, 412	<0.001	0.052	HS − PG: 13.61 [9.27, 17.95], *p* < 0.001; UG − PG: 11.87 [7.82, 15.92], *p* < 0.001 (HS − UG ns)
AEE	29.93 (6.63)	28.80 (6.63)	24.08 (7.80)	10.62	2, 412	<0.001	0.049	HS − PG: 5.85 [3.01, 8.69], *p* < 0.001; UG − PG: 4.72 [1.97, 7.47], *p* < 0.001 (HS − UG ns)
AES	13.71 (5.48)	13.55 (5.46)	10.94 (4.61)	3.85	2, 412	0.020	0.018	HS − PG: 2.77 [0.98, 4.56], *p* = 0.002; UG − PG: 2.61 [0.91, 4.31], *p* = 0.003 (HS − UG ns)
GA	12.80 (6.26)	12.36 (6.11)	7.82 (3.60)	9.80	2, 412	<0.001	0.045	HS − PG: 4.98 [3.39, 6.57], *p* < 0.001; UG − PG: 4.54 [3.10, 5.98], *p* < 0.001 (HS − UG ns)

Values are mean (SD). Welch's ANOVA reported when variances were unequal; otherwise one-way ANOVA. *Post hoc* tests were Games–Howell (unequal variances) or Tukey HSD (equal variances). Omnibus effect size is partial *η*^2^. The rightmost column lists significant pairwise differences as Δ = Mean difference (group1 − group2) with 95% CIsand *p*-values; “ns”, not significant. Tests two-tailed, *α* = 0.05; very small *p*-values reported as *p* < 0.001. EMAS, exercise motivation attitude scale; AFEAS, awareness for exercise addiction scale; NAT, negative attitudes & thoughts; PPH, positive perspective & health; PAH, physical appearance & health; AEE, awareness of emotional effects; AES, awareness of social effects; GA, general awareness.

Upon examining Table 4, it is observed that participants' levels of exercise motivation and awareness of exercise addiction significantly differ according to their physical activity frequency (*p* < 0.05). Individuals who engage in physical activity 1–2 days per week or 5 or more days per week have significantly higher EMAS total scores compared to those who are physically active less than once a week. Similarly, higher levels of physical activity are associated with increased motivation in the NAT, PPH, and PAH subdimensions. In terms of AFEAS scores, participants who exercise 1–2 days per week demonstrate significantly greater awareness levels than the other groups (*p* < 0.05). These findings suggest that regular physical activity may enhance both exercise motivation and awareness of exercise addiction.

**Table 4:** Physical-activity frequency differences (one-way ANOVA).

Measure	<1 day/week mean (SD)	1–2 days/week mean (SD)	3–4 days/week mean (SD)	≥5 days/week mean (SD)	*F* or *F*_Welch_	df1, df2	*p*	Partial *η*^2^	Significant *post hoc* contrasts (Δ [95% CI], *p*)
EMAS—Total	120.35 (19.89)	131.93 (16.32)	127.07 (19.85)	133.59 (8.82)	9.91	3, 411	<0.001	0.067	<1 − 1–2: −11.58 [−16.68, −6.48], *p* < 0.001; <1 − 3–4: −6.72 [−12.35, −1.09], *p* = 0.020; <1 − ≥5: −13.24 [−18.13, −8.35], *p* < 0.001; 1–2 − 3–4: 4.86 [0.47, 9.25], *p* = 0.030; 3–4 − ≥ 5: −6.52 [−10.65, −2.39], *p* = 0.002
NAT	45.44 (6.12)	47.41 (4.01)	46.94 (6.12)	47.93 (2.18)	3.68	3, 411	0.012	0.026	<1 − 1–2: −1.97 [−3.46, −0.48], *p* = 0.010; <1 − ≥ 5: −2.49[−3.94, −1.04], *p* = 0.001
PPH	40.72 (9.57)	45.16 (8.47)	43.22 (9.65)	45.79 (4.85)	5.91	3, 411	<0.001	0.041	<1 − 1–2: −4.44 [−6.95, −1.93], *p* < 0.001; <1 − ≥ 5: −5.07[−7.49, −2.65], *p* < 0.001; 3–4 − ≥ 5: −2.57 [−4.65, −0.49], *p* = 0.016
PAH	34.18 (8.19)	39.36 (6.14)	36.90 (8.80)	39.85 (3.57)	11.46	3, 411	<0.001	0.077	<1 − 1–2: −5.18 [−7.39, −2.97], *p* < 0.001; <1 − 3–4: −2.72[−5.13, −0.31], *p* = 0.025; <1 − ≥ 5: −5.67 [−7.64, −3.70], *p* < 0.001; 3–4 − ≥ 5: −2.95 [−5.06, −0.84], *p* = 0.006
AFEAS—Total	52.92 (16.39)	59.10 (15.80)	50.92 (14.51)	51.42 (12.53)	8.10	3, 411	<0.001	0.056	<1 − 1–2: −6.18 [−10.06, −2.30], *p* = 0.002; 1–2 − 3–4: 8.18 [4.43, 11.93], *p* < 0.001
AEE	27.32 (7.58)	30.02 (6.70)	27.63 (6.94)	30.00 (5.59)	4.70	3, 411	<0.001	0.033	<1 − 1–2: −2.70 [−4.68, −0.72], *p* = 0.007; 1–2 − 3–4: 2.39 [0.76, 4.02], *p* = 0.004; 3–4 − ≥ 5: −2.37 [−4.21, −0.53], *p* = 0.012
AES	13.44 (5.27)	14.73 (5.60)	12.67 (4.92)	11.57 (5.56)	6.35	3, 411	<0.001	0.044	1–2 − 3–4: 2.06 [1.31, 2.81], *p* < 0.001; 1–2 − ≥ 5: 3.16 [1.53, 4.79], *p* < 0.001; <1 − ≥ 5: 1.87[0.08, 3.66], *p* = 0.041
GA	12.15 (6.03)	14.35 (5.97)	10.62 (5.89)	9.84 (5.44)	13.02	3, 411	<0.001	0.087	1–2 − 3–4: 3.73 [2.31, 5.15], *p* < 0.001; 1–2 − ≥ 5: 4.51 [2.87, 6.15], *p* < 0.001; <1 − 1–2: −2.20 [−3.84, −0.56], *p* = 0.008; <1 − ≥ 5: 2.31 [0.43, 4.19], *p* = 0.016

Values are mean (SD). Welch's ANOVA reported when variances were unequal; otherwise one-way ANOVA. *Post hoc* tests were Games–Howell (unequal variances) or Tukey HSD (equal variances). Omnibus effect size is partial *η*^2^. The rightmost column lists significant pairwise differences as Δ = Mean difference (group1 − group2) with 95% CIsand *p*-values; “ns”, not significant. Tests two-tailed, *α* = 0.05; very small *p*-values reported as *p* < 0.001. EMAS, exercise motivation attitude scale; AFEAS, awareness for exercise addiction scale; NAT, negative attitudes & thoughts; PPH, positive perspective & health; PAH, physical appearance & health; AEE, awareness of emotional effects; AES, awareness of social effects; GA, general awareness.

Upon examining Table 5, no significant relationship was found between participants' age and their scores on EMAS, PPH, PAH, AFEAS Total, AEE, AES, or GA (*p* > .05). Age was negatively correlated with NAT (*r* = −0.17, *p* < 0.01); correlations with other subscales were non-significant. This suggests that as age increases, negative thoughts and feelings toward exercise tend to decrease.

**Table 5:** Correlations between age and EMAS/AFEAS scores.

Variable (*r* with age)	EMAS Total	NAT	PPH	PAH	AFEAS Total	AEE	AES	GA
Age (*n* = 415)	−0.05	−0.17	0.00	−0.02	0.01	0.01	0.00	0.06

Entries are Pearson's *r* (two-tailed); *n* = 415; *α* = 0.05. EMAS, exercise motivation attitude scale; AFEAS, awareness for exercise addiction scale; NAT, negative attitudes & thoughts; PPH, positive perspective & health; PAH, physical appearance & health; AEE, awareness of emotional effects; AES, awareness of social effects; GA, general awareness. Correlations were computed with listwise deletion.

Correlations among EMAS and AFEAS subscales are presented in Table 6. Correlations marked with * indicate significance at *p* < 0.05, and correlations marked with ** indicate significance at *p* < 0.01. Upon examining Table 6, a positive and significant relationship was found between overall exercise motivation and awareness of exercise addiction (*r* = 0.36, *p* < 0.01). These patterns support convergent validity—PPH and PAH correlate positively with weekly exercise frequency and AFEAS awareness (and, where applicable, AEE/AES/GA)—and discriminant validity, indicated by near-zero associations with unrelated demographics and modest inter-subscale correlations. At the subscale level, PPH (*r* = 0.40, *p* < 0.01) and PAH (*r* = 0.39, *p* < 0.01) showed moderate positive associations with AFEAS scores, whereas NAT was not significantly related to AFEAS. Positive and significant correlations were also observed between AEE, AES, GA and the PPH/PAH subscales of EMAS.

**Table 6:** Correlations among EMAS (total/subscales) and AFEAS (total/subscales).

Variables	1	2	3	4	5	6	7	8
1. EMAS Total	1.00	0.56**	0.93**	0.91**	0.36**	0.53**	0.21**	0.13**
2. NAT		1.00	0.33**	0.30**	0.01	0.12**	−0.05	−0.08
3. PPH			1.00	0.84**	0.40**	0.57**	0.23**	0.16**
4. PAH				1.00	0.39**	0.52**	0.24**	0.17**
5. AFEAS Total					1.00	0.76**	0.89**	0.87**
6. AEE						1.00	0.45**	0.39**
7. AES							1.00	0.85**
8. GA								1.00

The upper triangle and diagonal are displayed. EMAS, exercise motivation attitude scale; AFEAS, awareness for exercise addiction scale; NAT, negative attitudes & thoughts; PPH, positive perspective & health; PAH, physical appearance & health; AEE, awareness of emotional effects; AES, awareness of social effects; GA, general awareness.

* Significance: *p* < 0.05.

** Significance: *p* < 0.01.

## Discussion

This study aimed to investigate the relationship between individuals' awareness of exercise addiction and their motivation toward exercise, as well as how this relationship varies according to demographic variables, including gender, education level, age, and frequency of physical activity. The findings offer valuable insights into the coexistence of motivational attitudes and addiction awareness in different population groups, thereby contributing to the growing body of literature at the intersection of exercise psychology and behavioural health.

It observed a small-to-moderate positive association between health-oriented exercise motivation and awareness of exercise addiction. Interpreted cautiously in a cross-sectional design, this aligns with two plausible pathways: a protective correlate (autonomous motivation fostering reflective self-monitoring and timely boundary setting) and a risk-adjacent correlate (heightened vigilance among individuals more preoccupied with exercise). It considers both interpretations alongside the education- and frequency-related patterns and outlines designs to adjudicate between them (e.g., longitudinal tracking; qualitative probes of how “warning signs” are construed). The results indicate a moderate, positive relationship between overall exercise motivation and awareness of exercise addiction. This suggests that individuals who exhibit higher levels of motivation toward physical activity also tend to show greater cognitive awareness of the potential risks associated with excessive or compulsive exercise behaviours. In particular, positive and statistically significant correlations were found between PPH and PAH dimensions of motivation and the overall awareness of exercise addiction. These findings suggest that individuals motivated by health and appearance-related goals may also possess a higher degree of reflection and understanding about the limits and psychological implications of exercise (65–67). Similarly, regional evidence has shown that nutritional behaviors and dietary patterns significantly affect athletes' psychological balance and performance awareness (68). Findings indicate that targeted interventions can strengthen intrinsic motivation and promote active physical engagement (69). Notably, no significant correlation was found between NAT dimension and addiction awareness, suggesting that motivational deficits or negative beliefs about exercise do not necessarily correspond with increased self-awareness of problematic exercise tendencies (70–72).

When examining the influence of gender, the overall scores on exercise motivation and addiction awareness did not significantly differ between male and female participants. Higher NAT among women may reflect social-evaluative pressures and appearance norms documented in the literature, whereas men's slightly higher AES could relate to sport subcultures that normalise intensive training yet simultaneously heighten attention to social consequences (e.g., time conflicts). Effects were small, and sport type likely moderates these trends; future models should adjust for participation context (team/individual; competitive/recreational) and body-image pressure indices. However, women scored significantly higher on the NAT dimension, indicating that they may experience more emotional conflict or self-critical thoughts related to exercise (73, 74). While no significant gender-based differences emerged in awareness levels, the slightly higher scores among men in AES dimension could suggest that men are more attuned to how their exercise behaviors impact their social lives—potentially due to sport-oriented subcultures that normalize intensive training but may also reinforce social withdrawal (75–77).

In terms of educational background, the study found that undergraduate participants scored higher in both motivation and awareness compared to those in the postgraduate group, who reported significantly lower values across several dimensions. One interpretation is a resource/opportunity mechanism: undergraduates may have greater access to peer networks, campus facilities, and organised sport, which can both strengthen motivation and heighten awareness through exposure to risk communication in university settings. Conversely, lower scores among postgraduates could reflect time and role strain academic/professional demands compress time for reflective self-regulation and reduce participation in recreational activity. The higher awareness among high school graduates suggests a non-monotonic trajectory, potentially driven by different goal profiles (health/appearance vs. performance) and by communication channels (social media, school-based campaigns). (i) Cohort composition/confounding (age, employment) may account for part of the pattern; (ii) measurement coverage our attitudinal facets may be more sensitive to the kinds of goals prevalent in undergraduate populations; (iii) selection effects students who remain physically active during university might already be more reflective. Analytically, model education effects adjusted for age, sex, and exercise frequency; test education × frequency interactions; and examine sport type as a stratifier. Prospectively, use time-use covariates (work/study hours) and longitudinal tracking to determine whether changes in role strain predict shifts in motivation and awareness. However, awareness was greatest among high school participants, indicating that motivational and awareness patterns do not follow the same trajectory across educational levels. This inverse pattern between education level and the key variables may reflect the shifting priorities of individuals as they progress academically and professionally, such as increasing time constraints, a stronger academic identity over physical identity, or reduced participation in recreational physical activity (78–80). Conversely, high scores among undergraduates could be attributed to increased peer influence, exposure to fitness trends, and engagement in university sports activities.

The findings related to physical activity frequency revealed that individuals who exercise more regularly, especially those active 1–2 or ≥5 days per week, tend to report higher levels of both motivation and awareness compared to those who are physically active less than once a week. The pattern—motivation highest at ≥5 days/week while awareness peaks at 1–2 days/week—is compatible with an inverted-U in risk perception: limited but regular engagement may provide enough experiential feedback (fatigue, scheduling conflicts) to trigger reflection about limits, whereas very high frequency can coincide with normalisation within training subcultures, potentially dampening perceived risk despite high motivation. (i) Measurement reactivity: individuals training ≥5 days/week may construe “awareness” as routine caution rather than risk; (ii) unmeasured training load: intensity/duration (not captured by days/week) could better explain awareness; (iii) goal orientation differences (health/appearance/performance) may moderate the link. Test curvilinear models (quadratic/spline terms for frequency), add intensity and session duration covariates, and examine goal orientation as a moderator. For stronger inference, use ecological momentary assessment to couple day-level load with day-level awareness signals, and evaluate whether changes in load predict subsequent changes in awareness (lagged analyses). More specifically, motivation was highest among those exercising ≥5 days per week, while awareness peaked in the 1–2 days per week group. This supports the notion that greater behavioural engagement in exercise may foster not only stronger motivational patterns but also enhance individuals' self-monitoring capacity and recognition of healthy vs. compulsive behaviour (81–83). The association was particularly pronounced in the motivational subdimensions (PPH, PAH) and AEE and GA dimensions, indicating that physically active individuals may be more reflective of how exercise influences their emotional state and broader life quality (39, 42, 84). This could also be linked to experiential learning, as individuals encounter physical or psychological strain from high training loads, they may become more aware of the signs of imbalance.

Regarding age, the analysis did not show significant relationships between age and most subdimensions of exercise motivation and addiction awareness. However, a significant negative correlation was identified between age and the NAT dimension, implying that older individuals are less likely to report negative attitudes and thoughts toward exercise (85–87). Lower NAT with age is consistent with maturational gains in self-regulation and a shift toward intrinsic/health-maintenance goals. A survivorship account is also possible: individuals who maintain activity into later ages may already possess adaptive profiles. To distinguish age from cohort effects, longitudinal or age–period–cohort designs are needed. This finding is consistent with previous research suggesting that older adults tend to adopt a more balanced, intrinsic perspective on physical activity, prioritizing health maintenance and quality of life over performance or appearance (88–90). This also indicates that with age, individuals develop more sustainable motivational orientations and become less susceptible to guilt-driven or externally imposed motivations.

Finally, the correlation matrix revealed meaningful interrelationships between the subdimensions of both constructs. Within this context, the PPH–AEE association was moderate, whereas the PPH–AES and PPH–GA associations were small, indicating that individuals who maintain a positive, health-focused outlook on exercise are also more aware of its emotional and social risks when pursued excessively (91–93). This pattern suggests that cultivating positive exercise attitudes may function as a protective correlate against the development of unhealthy habits. Conversely, the absence of significant associations between NAT and awareness indicates that holding negative views toward exercise does not, by itself, enhance insight into exercise addiction and may instead reflect disengagement or limited self-reflection. Taken together, these interpretations are associational and should be treated as hypotheses pending longitudinal and experimental tests that can adjudicate among alternative mechanisms. Related regional studies among team athletes also highlighted the role of mood regulation and psychological preparedness in maintaining optimal performance (94). Moreover, evidence from sports officials shows that emotional regulation skills significantly influence performance consistency and psychological balance, underscoring the broader relevance of self-awareness in sports contexts (95).

### Limitations

Findings should be interpreted in light of sampling and design constraints. Although internal consistency and factorial fit were acceptable in this sample, further cross-cultural replication is needed to strengthen the generalisability of EMAS subscale interpretations. The cross-sectional, self-report design and online convenience sampling of predominantly university-affiliated young adults limit causal inference and external validity, and replication with more diverse/probability samples and objective behavioural indices is warranted. No *a priori* sample-size calculation was conducted; sensitivity analyses indicated adequate power for small-to-moderate effects but not for very small effects (59–61). Finally, multiple tests were conducted; no global multiplicity correction was applied beyond *post-hoc* procedures, so Type I error inflation remains possible.

### Implications and recommendations

The results of this study have several practical implications. First, promoting exercise awareness programs that target not only motivational enhancement but also knowledge about addiction risks can help individuals develop healthier and more sustainable exercise habits. Second, interventions targeting university populations, especially undergraduates, should acknowledge their higher motivation levels while also emphasizing the importance of self-regulation and maintaining a balance. Third, physical activity promotion efforts in public health and educational contexts should address motivational diversity, encouraging intrinsic goals while raising awareness about the psychological risks associated with excessive behaviour, particularly among those motivated by appearance or external validation.

Furthermore, future research should consider longitudinal designs to explore how changes in motivation influence the development of addiction awareness over time. Qualitative approaches could also deepen the understanding of subjective experiences and coping strategies among those who struggle to find balance in their exercise routines.

## Conclusion

This study investigated the relationship between individuals' motivation for exercise and their awareness of exercise addiction, with additional analyses conducted across demographic variables, including gender, age, educational background, and frequency of physical activity. The findings revealed a significant positive relationship between overall exercise motivation and awareness of exercise addiction, particularly among individuals motivated by health and physical appearance. Notably, no such relationship was observed with negative motivational attitudes, suggesting that positive motivational drivers may be more closely linked to self-reflection and awareness of behavioural limits.

The study also demonstrated that demographic variables play a nuanced role in shaping these constructs. While gender did not produce broad differences in motivation or awareness, women exhibited more negative emotional associations with exercise. Educational level was inversely related to both motivation and awareness, with undergraduate participants scoring higher than those at the postgraduate level. The frequency of physical activity emerged as a key differentiator—individuals who exercised more regularly displayed higher scores in both motivation and addiction awareness, reinforcing the role of behavioural engagement in fostering both commitment and self-regulation. In contrast, age was not broadly associated with the main variables, though a significant negative relationship was found between age and negative exercise attitudes.

In summary, the results suggest that fostering positive exercise motivations not only enhances engagement but also serves as a protective factor against compulsive or unhealthy exercise patterns. Awareness of exercise addiction appears to be more developed among those with consistent physical activity habits and health-oriented goals. These findings underscore the importance of promoting balanced and informed approaches to physical activity, particularly among young adults and university populations. Future interventions should focus on integrating motivational enhancement with educational components to promote both adherence and awareness, thereby supporting long-term behavioural sustainability and overall well-being.

## Data Availability

The raw data supporting the conclusions of this article will be made available by the authors, without undue reservation.

## References

[1] Dhuli K Naureen Z Medori MC Fioretti F Caruso P Perrone MA Physical activity for health. J Prev Med Hyg. (2022) 63(2 Suppl 3):E150–9. 10.15167/2421-4248/jpmh2022.63.2S3.2756 36479484 PMC9710390

[2] Qiu Y Fernández-García B Lehmann HI Li G Kroemer G López-Otín C Exercise sustains the hallmarks of health. J Sport Heal Sci. (2023) 12(1):8–35. 10.1016/j.jshs.2022.10.003 PMC9923435 36374766

[3] Hung ST Cheng YC Wu CC Su CH. Examining physical wellness as the fundamental element for achieving holistic well-being in older persons: review of literature and practical application in daily life. J Multidiscip Healthc. (2023) 16:1889–904. 10.2147/JMDH.S419306 37435298 PMC10329914

[4] Watson MC Lloyd J. Physical activity: manifold benefits for health and wellbeing. Br Med J. (2022) 376:o815. 10.1136/bmj.o815 35354590

[5] Singh B Olds T Curtis R Dumuid D Virgara R Watson A Effectiveness of physical activity interventions for improving depression, anxiety and distress: an overview of systematic reviews. Br J Sports Med. (2023) 57(18):1203–9. 10.1136/bjsports-2022-106195 36796860 PMC10579187

[6] Shilton T Bauman A Beger B Chalkley A Champagne B Elings-Pers M More people, more active, more often for heart health – taking action on physical activity. Glob Heart. (2024) 19(1):42. 10.5334/gh.1308 38708404 PMC11067976

[7] Dasgupta P. The benefits of regular exercise. BJU Int. (2020) 125(1):1. 10.1111/bju.14966 31901012

[8] Valenzuela PL Ruilope LM Santos-Lozano A Wilhelm M Kränkel N Fiuza-Luces C Exercise benefits in cardiovascular diseases: from mechanisms to clinical implementation. Eur Heart J. (2023) 44(21):1874–89. 10.1093/eurheartj/ehad170 37005351

[9] Oral O Rezaee Z Thapa P Nomikos GN Enser M. A comprehensive review of the metabolic and psychophysiological effect of regular exercise on healthy life. Glob Sport Sci. (2024) 3:26–30. 10.62836/gss.v3i1.170

[10] Mallardo M Daniele A Musumeci G Nigro E. A narrative review on adipose tissue and overtraining: shedding light on the interplay among adipokines, exercise and overtraining. Int J Mol Sci. (2024) 25(7):4089. 10.3390/ijms25074089 38612899 PMC11012884

[11] Meng Q Su CH. The impact of physical exercise on oxidative and nitrosative stress: balancing the benefits and risks. Antioxidants. (2024) 13(5):573. 10.3390/antiox13050573 38790678 PMC11118032

[12] Lichtenstein MB Melin AK Szabo A Holm L. The prevalence of exercise addiction symptoms in a sample of national level elite athletes. Front Sport Act Living. (2021) 3:635418. 10.3389/fspor.2021.635418 PMC8222598 34179773

[13] Weinstein A Szabo A. Exercise addiction: a narrative overview of research issues. Dialogues Clin Neurosci. (2023) 25(1):1–13. 10.1080/19585969.2023.2164841 36698618 PMC9869993

[14] Godoy-Izquierdo D Navarrón E López-Mora C González-Hernández J. Exercise addiction in the sports context: what is known and what is yet to be known. Int J Ment Health Addict. (2023) 21(2):1057–74. 10.1007/s11469-021-00641-9

[15] Colledge F Meyer M. Exercise addiction – status, identification and treatment. Praxis. (2022) 111(6):317–21. 10.1024/1661-8157/a003875 35473333

[16] Orhan BE Alkasasbeh W Karaçam A. Understanding exercise addiction in women: exploring the impact of sociodemographic variables. Montenegrin J Sport Sci Med. (2025) 14(2):3–10. 10.26773/mjssm.250904

[17] Lampe EW Schaumberg K Kolar D Coniglio K Cooper M Chapa DAN Working out measurement overlap in the assessment of maladaptive exercise. Int J Eat Disord. (2024) 57(3):558–67. 10.1002/eat.24127 38221645 PMC10947899

[18] Khazaal Y Chappuis L Maccaferri GE Gremeaux V. Addiction à l’exercice. Rev Med Suisse. (2024) 20(882):1354–9. 10.53738/REVMED.2024.20.882.1354 39021105

[19] Wang Y Hua G Liu W Wan C Hao M Zhang M. Exercise addiction in college students: the impact of body dissatisfaction, stress, physical activity and gender. Front Psychiatry. (2025) 16:1546192. 10.3389/fpsyt.2025.1546192 40071282 PMC11893607

[20] Chhabra B Granziol U Griffiths MD Zandonai T Landolfi E Solmi M Prevalence of the risk of exercise addiction based on a new classification: a cross-sectional study in 15 countries. Int J Ment Health Addict. (2024) 22:1–22. 10.1007/s11469-024-01322-z

[21] Dinardi JS Egorov AY Szabo A. The expanded interactional model of exercise addiction. J Behav Addict. (2021) 10(3):626–31. 10.1556/2006.2021.00061 34524973 PMC8997218

[22] Guo S Kamionka A Xue Q Izydorczyk B Lipowska M Lipowski M. Body image and risk of exercise addiction in adults: a systematic review and meta-analysis. J Behav Addict. (2025) 14(1):39–54. 10.1556/2006.2024.00085 39912824 PMC11974424

[23] Cuesta-Zamora C Ricarte JJ Ros L Latorre JM Plateau C. The role of intolerance of uncertainty and anxiety on compulsive exercise in adolescents. Anxiety Stress Coping. (2023) 36(5):649–60. 10.1080/10615806.2023.2188204 36943399

[24] Cosh SM McNeil DG Tully PJ. Compulsive exercise and its relationship with mental health and psychosocial wellbeing in recreational exercisers and athletes. J Sci Med Sport. (2023) 26(7):338–44. 10.1016/j.jsams.2023.05.006 37296060

[25] Nicholls K Ogden J. Rationalising a spectrum of problematic exercise: a qualitative study. J Health Psychol. (2025) 30(8):1839–54. 10.1177/13591053241274471 39269032 PMC12227822

[26] Caponnetto P Casu M Amato M Cocuzza D Galofaro V La Morella A The effects of physical exercise on mental health: from cognitive improvements to risk of addiction. Int J Environ Res Public Health. (2021) 18(24):13384. 10.3390/ijerph182413384 34948993 PMC8705508

[27] Gori A Topino E Griffiths MD. Protective and risk factors in exercise addiction: a series of moderated mediation analyses. Int J Environ Res Public Health. (2021) 18(18):9706. 10.3390/ijerph18189706 34574631 PMC8467293

[28] Gjestvang C Bratland-Sanda S Mathisen TF. Compulsive exercise and mental health challenges in fitness instructors; presence and interactions. J Eat Disord. (2021) 9(1):107. 10.1186/s40337-021-00446-0 34493315 PMC8422740

[29] Landolfi E. An investigation of exercise behaviour patterns amongst varsity athletes. J Concurr Disord. (2022) 1–12. 10.54127/HHDT5954

[30] Rodrigues F Cid L Faustino T Monteiro D. The situational motivation scale in the exercise context: construct validity, factor structure, and correlational analysis. Curr Psychol. (2023) 42(6):4811–20. 10.1007/s12144-021-01824-2

[31] Meyer JP Espinoza JA Vaters C Anderson BK Beletski LV. Motivational mindsets versus reasons for action: implications for the dimensionality debate in self-determination theory. Motiv Emot. (2022) 46(4):486–507. 10.1007/s11031-022-09958-x

[32] De Moraes Ovando RG Morais dos Santos LL Marques Gomes Bertolini SM. Motivational profile for physical exercise: a retrospective cohort. Retos. (2023) 50:8–14. 10.47197/retos.v50.99435

[33] Nam TT Le CN Phu DH Stanikzai MH Shohaimi S Dadras O Assessment of self-determined motivation in exercise: a systematic review and meta-analysis. J Hum Earth Future. (2023) 4(2):241–56. 10.28991/HEF-2023-04-02-08

[34] Alkasasbeh WJ Akroush SH. Sports motivation: a narrative review of psychological approaches to enhance athletic performance. Front Psychol. (2025) 16:1645274. 10.3389/fpsyg.2025.1645274 40831475 PMC12358434

[35] Deci EL Ryan RM. The “what” and “why” of goal pursuits: human needs and the self-determination of behavior. Psychol Inq. (2000) 11(4):227–68. 10.1207/S15327965PLI1104_01

[36] Ryan RM Deci EL. Self-determination theory. In: Encyclopedia of Quality of Life and Well-Being Research. Cham: Springer (2024). p. 6229–35.

[37] Rodrigues F Jacinto M Couto N Monteiro D Monteiro AM Forte P Motivational correlates, satisfaction with life, and physical activity in older adults: a structural equation analysis. Medicina. (2023) 59(3):599. 10.3390/medicina59030599 36984600 PMC10052721

[38] Arashi M Webster CA Mîndrila D Duda JL Stankić D Perić D Associations between intrinsic motivation, exercise attitudes, physical activity, and mental health in young adolescents: an integrated motivational perspective. Res Q Exerc Sport. (2025) 96(3):513–22. 10.1080/02701367.2024.2446545 39883544

[39] Carraça EV Encantado J Battista F Beaulieu K Blundell JE Busetto L Effect of exercise training on psychological outcomes in adults with overweight or obesity: a systematic review and meta-analysis. Obes Rev. (2021) 22(S4):e13261. 10.1111/obr.13261 33960106 PMC8365728

[40] Ghayour Baghbani SM Arabshahi M Saatchian V. The impact of exercise interventions on perceived self-efficacy and other psychological outcomes in adults: a systematic review and meta-analysis. Eur J Integr Med. (2023) 62:102281. 10.1016/j.eujim.2023.102281

[41] Çakın G Juwono ID Potenza MN Szabo A. Exercise addiction and perfectionism: a systematic review of the literature. Curr Addict Rep. (2021) 8(1):144–55. 10.1007/s40429-021-00358-8

[42] Vosadi E Hashemi Fard ES Mirakhori Z Borjian Fard M. The impact of exercise training on psychological outcomes, body composition, and quality of life in overweight or obese adults: a systematic review and meta-analysis of randomized controlled trials. Biol Res Nurs. (2025) 27(3):464–86. 10.1177/10998004241313332 39792027

[43] Chen Y Li J Wang X Chen J Fan H. Are college perfectionists more likely to be addicted to exercise? The promotion effect of psychological needs and motivation. Int J Sport Exerc Psychol. (2025) 1–20. 10.1080/1612197X.2025.2481431

[44] Vítková T Rusnáková K Mudrák J. Personality predictors of exercise addiction in competitive sport. J Sports Sci. (2025) 43(9):865–74. 10.1080/02640414.2025.2477922 40091662

[45] Smith MA Kingston S. Demographic, attitudinal, and social factors that predict pro-environmental behavior. Sustain Clim Chang. (2021) 14(1):47–54. 10.1089/scc.2020.0063

[46] Muthukumar P Krishnakumar E. Behavioral biases towards investment decisions on demographic characteristics. Int J Sci Res. (2024) 13(4):1767–9. 10.21275/SR24426102058

[47] de Valle MK Gallego-García M Williamson P Wade TD. Social media, body image, and the question of causation: meta-analyses of experimental and longitudinal evidence. Body Image. (2021) 39:276–92. 10.1016/j.bodyim.2021.10.001 34695681

[48] Popat A Tarrant C. Exploring adolescents’ perspectives on social media and mental health and well-being – a qualitative literature review. Clin Child Psychol Psychiatry. (2023) 28(1):323–37. 10.1177/13591045221092884 35670473 PMC9902994

[49] Viinikainen J Bryson A Böckerman P Kari JT Lehtimäki T Raitakari O Does better education mitigate risky health behavior? A Mendelian randomization study. Econ Hum Biol. (2022) 46:101134. 10.1016/j.ehb.2022.101134 35354116

[50] Murakami K Kuriyama S Hashimoto H. Economic, cognitive, and social paths of education to health-related behaviors: evidence from a population-based study in Japan. Environ Health Prev Med. (2023) 28:9. 10.1265/ehpm.22-00178 36709974 PMC9884565

[51] Martínez-Sánchez SM Martínez-Sánchez LM Martínez-García C. Gender differences in barriers to physical exercise among university students studying physical activity and sports sciences. Health Educ J. (2024) 83(2):192–204. 10.1177/00178969231226383

[52] Oliver-López A Vega-Díaz M Sáenz A González-García H. Relationships among physical self-concept profiles, orthorexia nervosa, and exercise addiction in crossfitters. J Clin Sport Psychol. (2024) 1:1–18. 10.1123/jcsp.2024-0010

[53] Kalayasiri R Rattanawijarn C. Demographics and physical and mental health of clients at a sports center with and without exercise addiction. PeerJ. (2025) 13:e19002. 10.7717/peerj.19002 40151458 PMC11949105

[54] Soraci P Pisanti R Servidio RC Lo Destro C Melchiori FM La Rocca A Does self-esteem mediate the relationship between stress and exercise addiction? Psychol Hub. (2024) 41(3):23–30. 10.13133/2724-2943/18355

[55] Soraci P Szabo A Vegni N Prestano C Guaitoli E Di Bernardo C Psychometric evaluation of the Italian revised exercise addiction inventory (EAI-R) among Italian speaking exercisers: confirmatory factor analysis. Psychol Hub. (2023) 40(2):31–40. 10.13133/2724-2943/17987

[56] Chabbra B Bartos A Soós I Ruíz-Barquín R de la Vega R Szabo A. Passion, perfectionism, and sports commitment as predictors of exercise addiction. Eur J Sport Sci. (2025) 25(11):e70068. 10.1002/ejsc.70068 41108529 PMC12535194

[57] Orhan BE Alkasasbeh W Karaçam A Govindasamy K. Exploring motivation and enjoyment as key determinants of sustained physical activity across diverse demographics. Sport Mont. (2025) 23(2):63–9. 10.26773/smj.250610

[58] Fraenkel JR Wallen NE. How to Design and Evaluate Research in Education. New York, New York: McGraw-Hill Higher Education (2009).

[59] Faul F Erdfelder E Buchner A Lang AG. Statistical power analyses using G*power 3.1: tests for correlation and regression analyses. Behav Res Methods. (2009) 41(4):1149–60. 10.3758/BRM.41.4.1149 19897823

[60] Cohen J. Statistical Power Analysis for the Behavioral Sciences. New York: Routledge (2013).

[61] Lakens D. Calculating and reporting effect sizes to facilitate cumulative science: a practical primer for t-tests and ANOVAs. Front Psychol. (2013) 4:863. 10.3389/fpsyg.2013.00863 24324449 PMC3840331

[62] Büyüköztürk Ş Akgün ÖE Demirel F Karadeniz Ş Çakmak EK. Bilimsel araştırma yöntemleri (2015).

[63] Demir GT Cicioğlu Hİ. Egzersiz Bağimliliğina İlişkin Farkindalik Ölçeği (EBIFÖ): Geçerlik Ve Güvenirlik Çalişmasi. Spor Performans Araşt Derg. (2022) 13(1):1–17. 10.17155/omuspd.1065498

[64] Caz Ç. Egzersiz Motivasyonu Tutum Ölçeği: Ölçek Geliştirme, Geçerlik ve Güvenirlik Çalışması. Siirt Üniv Sos Bilim Enst Derg. (2024) 12(1):1–12. 10.53586/susbid.1467978

[65] Earl SR. Global and appearance-contingent self-esteem: associations with health and attractiveness exercise reasons. Psychol Sport Exerc. (2023) 65:102345. 10.1016/j.psychsport.2022.102345 37665828

[66] Swann C Wagner D Clarke MM Goddard SG McKeon G Rosenbaum S Is there a need for mental health informed goal setting in physical activity? Ment Health Phys Act. (2024) 27:100648. 10.1016/j.mhpa.2024.100648

[67] Mahmood S. Analyzing the cognitive experiences of individuals linked to regular exercise at the gym: an interpretative phenomenological analysis (IPA). Migr Lett. (2024) 21(S10):611–20. 10.59670/ml.v21iS10.10546

[68] AlKasasbeh WJ Amawi AT. Impact of eating habits on the psychological state of Jordanian athletes: a descriptive study. Food Sci Technol. (2023) 11(3):168–81. 10.13189/fst.2023.110305

[69] Alkasasbeh WJ Alawamleh T Aloran H Farash T Orhan BE. The impact of mobile-assisted swimming applications on intrinsic motivation and fear reduction in aquatic environments among students in the swimming course. Front Sport Act Living. (2024) 6:1496733. 10.3389/fspor.2024.1496733 PMC11655226 39703546

[70] Seymour J Pratt G Patterson S Korman N Rebar A Tillston S Changes in self-determined motivation for exercise in people with mental illness participating in a community-based exercise service in Australia. Health Soc Care Community. (2022) 30(5):e1611–24. 10.1111/hsc.13588 34614232

[71] Turner MJ Miller A Youngs H Barber N Brick NE Chadha NJ “I must do this!”: A latent profile analysis approach to understanding the role of irrational beliefs and motivation regulation in mental and physical health. J Sports Sci. (2022) 40(8):934–49. 10.1080/02640414.2022.2042124 35220909

[72] Turner M Miller A Youngs H. The role of irrational beliefs and motivation regulation in worker mental health and work engagement: a latent profile analysis. Baker R, editor. PLoS One. (2022) 17(8):e0272987. 10.1371/journal.pone.0272987 35969527 PMC9377577

[73] Huellemann KL Pila E Gilchrist JD Nesbitt AE Sabiston CM. Body-related self-conscious emotions and reasons for exercise: a latent class analysis. Body Image. (2021) 38:127–36. 10.1016/j.bodyim.2021.03.016 33848697

[74] Sabiston CM Vani MF Murray RM. Body-related self-conscious emotions in sport and exercise. In: Chatzisarantis N, Hagger M, editors. Motivation and Self-Regulation in Sport and Exercise. New York: Routledge (2021). p. 62–77.

[75] Dawson N Hammer JH. No pain, no gains: conformity to masculine norms, body dissatisfaction, and exercise dependence. Psychol Men Masc. (2020) 21(3):430–40. 10.1037/men0000243

[76] Kim D Park J Park B. How do leisure activities impact leisure domain and life domain satisfaction and subjective well-being? Int J Tour Res. (2024) 26(1). 10.1002/jtr.2618

[77] Mansour K Greenwood CJ Francis LM Misuraca G Marabel-Whitburn K Olsson CA The value of social networks for men: concurrent and prospective associations with psychological wellbeing. BMC Psychol. (2025) 13(1):142. 10.1186/s40359-025-02467-9 39980014 PMC11843785

[78] Barbosa A Whiting S Simmonds P Scotini Moreno R Mendes R Breda J. Physical activity and academic achievement: an umbrella review. Int J Environ Res Public Health. (2020) 17(16):5972. 10.3390/ijerph17165972 32824593 PMC7460146

[79] Cura SN. Constraints on student participation and skill development in physical education. Int J Res Publ. (2024) 153(1):23–33. 10.47119/IJRP1001531720246953

[80] Teuber M Leyhr D Sudeck G. Physical activity improves stress load, recovery, and academic performance-related parameters among university students: a longitudinal study on daily level. BMC Public Health. (2024) 24(1):598. 10.1186/s12889-024-18082-z 38402396 PMC10893600

[81] Teo JL Zheng Z Bird SR. Identifying the factors affecting ‘patient engagement’ in exercise rehabilitation. BMC Sports Sci Med Rehabil. (2022) 14(1):18. 10.1186/s13102-022-00407-3 35130940 PMC8819209

[82] André N Grousset M Audiffren M. A behavioral perspective for improving exercise adherence. Sports Med Open. (2024) 10(1):56. 10.1186/s40798-024-00714-8 38763991 PMC11102891

[83] Gao W Chen J Tu Z Li M. Correlational research on college students’ physical exercise behavior, academic engagement, and self-efficacy. Front Psychol. (2025) 16:1428365. 10.3389/fpsyg.2025.1428365 39963677 PMC11830674

[84] Mu FZ Liu J Lou H Zhu WD Wang ZC Li B. Influence of physical exercise on negative emotions in college students: chain mediating role of sleep quality and self-rated health. Front Public Heal. (2024) 12:1402801. 10.3389/fpubh.2024.1402801 PMC11100322 38765486

[85] Cavallini MF Tredway A Covan AJ Dyck DJ. Elderly individuals have similar attitudes towards physical activity and exercise as the young and middle aged, but are less likely to seek companionship or gym memberships. J Fam Med Dis Prev. (2022) 8(1):1–10. 10.23937/2469-5793/1510148

[86] Tsai TH Wong AM Lee HF Tseng KC. A study on the motivation of older adults to participate in exercise or physical fitness activities. Sustainability. (2022) 14(10):6355. 10.3390/su14106355

[87] Chen Y Holahan C Holahan C. Attitudes toward aging, physical activity, and functional limitations ten years later in middle and older adults. Innov Aging. (2020) 4(Supplement_1):913. 10.1093/geroni/igaa057.3356

[88] Pinheiro MB Oliveira JS Baldwin JN Hassett L Costa N Gilchrist H Impact of physical activity programs and services for older adults: a rapid review. Int J Behav Nutr Phys Act. (2022) 19(1):87. 10.1186/s12966-022-01318-9 35836187 PMC9284866

[89] Meredith SJ Cox NJ Ibrahim K Higson J McNiff J Mitchell S Factors that influence older adults’ participation in physical activity: a systematic review of qualitative studies. Age Ageing. (2023) 52(8):afad145. 10.1093/ageing/afad145 37595070 PMC10438214

[90] Schwartz BD Liu H MacDonald EE Mekari S O’Brien MW. Impact of physical activity and exercise training on health-related quality of life in older adults: an umbrella review. GeroScience. (2025) 47(3):2879–93. 10.1007/s11357-024-01493-6 39754009 PMC12181525

[91] Curreri AJ Farchione TJ Sauer-Zavala S Barlow DH. Mindful emotion awareness facilitates engagement with exposure therapy: an idiographic exploration using single case experimental design. Behav Modif. (2022) 46(1):36–62. 10.1177/0145445520947662 32752883

[92] Sánchez-López MT Fernández-Berrocal P Gómez-Leal R Megías-Robles A. Does emotional intelligence have the same role in each risk behaviour? Eur Psychiatry. (2022) 65(S1):S688–9. 10.1192/j.eurpsy.2022.1773

[93] González García JC Lozano Pineda C Cuartas Díaz M Torres-Barreto ML. Game-based exercise focused on emotional intelligence. Reg Cient. (2023) 2:1–9. 10.58763/rc202365

[94] Akroush S Alkasasbeh W Sha’lan M Abdi E Khatatbeh M. Psychological mood patterns among Jordanian handball players. Retos. (2025) 62:285–94. 10.47197/retos.v62.109407

[95] Karaçam A Orhan BE Alkasasbeh WJ Canlı U Adıgüzel NS. The role of emotional regulation in refereeing performance: the case of basketball. Phys Educ Theory Methodol. (2025) 25(5):1186–92. 10.17309/tmfv.2025.5.18

