# Supplementary material for: Awareness of exercise addiction and exercise motivation attitudes: a cross-sectional study
Source: Front Sports Act Living. 2025 Nov 28;7:1691151. doi: 10.3389/fspor.2025.1691151 (PMC12698601; doi:10.3389/fspor.2025.1691151)
Supplement: Supplementary file 1 [file Datasheet1.pdf]

Appendix/Table S1. Exercise Motivation Attitude Scale (EMAS) subdimensions: overview, example items, scoring, and psychometrics

Subdimension	Conceptual focus (SDT mapping)	Example item (paraphrased)†	Items (n)	Response scale	Scoring (direction / reversals)	Reliability — original (Cronbach's α)	AVE — original	Factor loadings — original (range)
**NAT — Negative Attitudes & Thoughts**	Controlled/amotivation cues; negative cognitions about exercise	"Sometimes exercise makes me feel uneasy." / "I think exercise is a waste of time."	7	1–7 (Strongly Disagree → Strongly Agree)	Reverse-score all NAT items (higher raw = more negative attitudes; reverse before computing subscale mean)	0.792	0.528	0.763–0.923
**PPH — Positive Perspective & Health**	Autonomous, health/well-being focus; positive affective/cognitive appraisals	"I exercise to protect my mental health." / "Exercise lifts my motivation."	7	1–7	Higher = more health/well-being-oriented motives	0.890	0.706	0.660–0.821
**PAH — Physical Appearance & Health**	Appearance-, control-, and health-maintenance motives	"I exercise to maintain a healthy/fit body." / "I want to avoid illness by exercising."	6	1–7	Higher = more appearance/control/health maintenance motives	0.933	0.711	0.705–0.815

Scale total: 20 items; overall α = 0.914; three-factor solution explains 70.092% of variance (NAT 38.548%, PPH 23.797%, PAH 7.747%). Confirmatory factor analysis (separate sample) showed $\chi^2/df = 2.640$, GFI = 0.861, IFI = 0.937, TLI = 0.927, CFI = 0.937, NFI = 0.902, RMSEA = 0.079, supporting the three-factor structure.

†Example items are paraphrased from the Turkish instrument to respect licensing. If the journal permits verbatim reproduction, you may replace paraphrases with the exact stems listed in the original (see EMAS article, pp. 10–12).

Not. EMAS = Exercise Motivation Attitude Scale; NAT = Negative Attitudes & Thoughts; PPH = Positive Perspective & Health; PAH = Physical Appearance & Health; AVE = Average Variance Extracted.
